# Psychometric Properties and Measurement Invariance of the Awareness of Age-Related Change Short Form in Older Adult Samples From Taiwan and Germany

**DOI:** 10.1093/geront/gnae086

**Published:** 2024-06-29

**Authors:** Han-Yun Tseng, Chi-Shin Wu, Chun-Yi Lee, I-Chien Wu, Hsing-Yi Chang, Chih-Cheng Hsu, Chao Agnes Hsiung, Roman Kaspar, Hans-Werner Wahl, Manfred Diehl

**Affiliations:** Department of Human Development and Family Studies, Colorado State University, Fort Collins, Colorado, USA; National Center for Geriatrics and Welfare Research, National Health Research Institutes, Zhunan, Miaoli County, Taiwan; Department of Psychiatry, National Taiwan University Hospital, Yunlin, Taiwan; Institute of Population Health Sciences, National Health Research Institutes, Miaoli County, Taiwan; Institute of Population Health Sciences, National Health Research Institutes, Miaoli County, Taiwan; Institute of Population Health Sciences, National Health Research Institutes, Miaoli County, Taiwan; National Center for Geriatrics and Welfare Research, National Health Research Institutes, Zhunan, Miaoli County, Taiwan; Institute of Population Health Sciences, National Health Research Institutes, Miaoli County, Taiwan; Institute of Population Health Sciences, National Health Research Institutes, Miaoli County, Taiwan; Charlotte Fresenius University of Psychology, Cologne, Germany; Network Aging Research & Institute of Psychology, Heidelberg University, Heidelberg, Germany; Department of Human Development and Family Studies, Colorado State University, Fort Collins, Colorado, USA

**Keywords:** Attitudes and perception toward aging/aged, Awareness of age-related changes (AARC), Healthy Aging Longitudinal Study in Taiwan (HALST), Measurement, Psychometrics

## Abstract

**Background and Objectives:**

This study examined the psychometric properties and measurement invariance of the 10-item Awareness of Age-Related Change Short Form (AARC-SF) questionnaire in a Chinese-speaking sample of older adults in Taiwan.

**Research Design and Methods:**

Data from 292 participants (*M*_age_ = 77.64 years) in the Healthy Aging Longitudinal Study in Taiwan cohort were used for Study 1, whereas data from young-old adult samples in Germany were used for Study 2.

**Results:**

Study 1 showed that the AARC-SF had satisfactory reliability and validity for assessing adults’ AARC in Taiwan. Analyses confirmed the 2-factor structure of AARC-gains and AARC-losses. Study 2 demonstrated strong measurement invariance across men and women, whereas direct comparisons of the item scores between young-old adults and old-old adults need to be made with caution. Noninvariance of loadings indicated that certain items were more closely linked to AARC-gains and AARC-losses in Taiwan than in Germany. Noninvariance of intercepts suggested potential biases in comparing item scores between Taiwanese and German older adults.

**Discussion and Implications:**

The AARC-SF emerged as a reliable and valid instrument for capturing positive and negative subjective aging experiences among Taiwanese older adults. However, it is noteworthy that some items on the AARC-SF may solicit different responses from individuals of different ages and different countries of origin, requiring caution with age group and cross-cultural comparisons.

The term ‘subjective views of aging’ (SVOA) has emerged as pivotal in understanding individuals’ subjective experience of aging, encompassing attitudes, beliefs, and feelings toward growing older (Diehl et al., 2014; Palgi et al., 2019; Wurm et al., 2017). SVOA serve as significant predictors of health and well-being, as evidenced by several systematic reviews and meta-analyses (e.g., Westerhof et al., 2014, 2023). Whereas SVOA have been extensively studied in many Western countries, research in Eastern countries remains relatively scarce. In this pursuit, the manuscript focuses on the importance of valid measurement tools to elucidate the development and changes of individuals’ SVOA across adulthood, accounting for potential variations by gender, age, and country.

## Measures of Subjective Views of Aging

Traditionally, individuals’ SVOA have been assessed with a single item question: “How old do you feel most of the time” (i.e., felt age; Barrett, 2003), which was originally developed to complement other measures of age (e.g., chronological age, biological age). SVOA has also been frequently assessed with the unidimensional, five-item Attitudes Toward Own Aging (ATOA) scale, a subscale of the Philadelphia Geriatric Center Morale Scale (Lawton, 1975). This scale asks about individuals’ global evaluation of their current life situation, including whether they feel their life is better compared to their younger years. Felt age and ATOA represent unidimensional measures of SVOA, and both indicators are important in understanding the relevance of SVOA for developmental outcomes beyond chronological age (Barrett, 2003).

Although these unidimensional SVOA measures are parsimonious and powerful in predicting developmental outcomes, one of the major shortcomings pointed out by Diehl and colleagues (2015) is the lack of explicit references to personal and behavior-specific aging experiences that may vary across life domains. In light of this, Diehl and Wahl (2010) introduced the concept of the *Awareness of Age-Related Change* (AARC) into the literature to increase researchers’ understanding of the specific aspects of individuals’ SVOA that influence health and well-being in later life.

## Awareness of Age-Related Change: A Multidimensional Approach

By definition, AARC refers to a person’s state of awareness “that his or her behavior, level of performance, or way of experiencing life has changed as a consequence of having grown older” (Diehl & Wahl, 2010, p. 342). In other words, adults’ AARC is a product of physical, cognitive, interpersonal, and social processes that involve personal and interpersonal experiences and individuals’ beliefs and expectations regarding aging and old age as a life stage. The construct of AARC, therefore, attempts to unpack what age-related experiences are involved when individuals contemplate their felt age and describe their attitudes toward their own aging.

Another feature of the AARC construct is that it is conceptually rooted in lifespan developmental theory (Baltes, 1987). The AARC concept includes both age-related gains and losses and explicitly focuses on changes in key behavioral domains. Therefore, AARC complements earlier conceptualizations of SVOA and contributes to the burgeoning advocacy for multidimensional approaches to measuring SVOA (e.g., the AgeCog Scales; Steverink et al., 2001; the Attitudes to Aging Questionnaire; Laidlaw et al., 2007) by encompassing both perceived gain- and loss-related changes in several behavioral domains that inform individuals’ global ratings of SVOA (Diehl et al., 2021).

## Development and Validation of the Awareness of Age-Related Changes Short Form

The development of the AARC questionnaire began with a 189-item version (Brothers et al., 2019; Study 1), with parallel forms in German and English, to comprehensively capture SVOA across the five specified behavioral domains (i.e., health and physical functioning, cognitive functioning, interpersonal relations, social–cognitive and social–emotional functioning, and lifestyle and engagement). In the process of psychometric refinement, the number of items was reduced to a more parsimonious 50-item version (Brothers et al., 2019; Study 2). To make the AARC more suitable for use in large-scale surveys with community-dwelling adults, the 10-item AARC Short Form (AARC-SF; Kaspar et al., 2019) was developed to assess adults’ positive and negative SVOA across the five behavioral domains.

Since its availability, the psychometric properties of the AARC-SF have been validated in American, British, and German community-dwelling adults aged 40 and older (Kaspar et al., 2019; Sabatini et al., 2020). Research has confirmed that a two-factor structure of the AARC-SF (i.e., AARC-gains and AARC-losses) outperformed a one-factor model (Kaspar et al., 2019; Sabatini et al., 2020). In these adult samples, the AARC-SF also demonstrated good reliability (Kaspar et al., 2019), supporting the argument that positive and negative aging experiences coexist in adults’ SVOA.

In terms of validity, a recent meta-analysis indicated small but significant associations between AARC and physical and mental well-being (Sabatini et al., 2020), supporting its criterion validity. AARC has also been found to uniquely account for a significant amount of variance in adults’ health and well-being, even after adjusting for global evaluations of SVOA such as felt age and ATOA (Brothers et al., 2017). Essentially, these results support the questionnaire’s discriminant validity with other established SVOA measures when it comes to predicting health and well-being in middle-aged and older adults.

## Measurement Invariance Across Gender, Age, and Country

Measurement invariance (MI) refers to the comparability of measured construct(s) across conditions such as time, gender and age groups, or individuals living in different countries. The absence of MI threatens meaningful comparisons across groups and may therefore make conclusions based on pooled data questionable. In light of this, prior research found that the 10 items in the AARC-SF contributed to the latent factors of AARC-gains and AARC-losses to a similar degree across groups of gender, age, educational levels, and marital status (i.e., metric invariance; Kaspar et al., 2019; Sabatini et al., 2020). However, scalar invariance where item intercepts or item difficulties were assumed to be equal did not hold between young-old (age 65–80 years) and old-old adults (age 80 and older; Kaspar et al., 2019), suggesting that comparisons of observed composite scores of AARC-gains and AARC-losses across different age groups should be made with caution.

In terms of different countries, the psychometric properties of the AARC-SF were established in Brazil (Neri et al., 2021), Norway (Testad et al., 2022), and South Korea (Nam & Kim, 2021). Despite their unique cultural, language, and societal contexts, all three studies found that the AARC-SF demonstrated good internal consistency, test-retest reliability, and construct validity. These studies also supported the best fit for a two-factor structure of the AARC-SF, with one factor assessing age-related losses and the other factor measuring age-related gains. Overall, these studies provided some evidence supporting the country-specific applicability and usefulness of AARC-SF in understanding age-related changes in diverse adult populations.

However, evidence on MI across countries is limited. It is important to note that full metric MI or scalar MI across countries may be difficult to achieve. Instead, it may be more realistic to expect a certain degree of measurement noninvariance. Such noninvariance may provide valuable information to what extent AARC in certain behavioral domains may be explained by culture-specific differences in shared values, beliefs, symbols, and the meaning of aging. In practical terms, valid comparison of AARC scores across nations and cultures can only be made if MI is supported.

## The Present Study

This study aimed to validate the Chinese version of the AARC-SF for use in Taiwan and investigate MI across gender, age groups, and countries. Study 1 examined (a) the item characteristics (e.g., item difficulty); (b) the internal consistency and reliability of the scale; (c) the scale’s factor structure; and (d) the scale’s convergent, discriminant, and criterion validity. Study 2 explored whether the same factor loading pattern (metric invariance) and item means (scalar invariance) held for (a) men and women, (b) young-old (aged 65–80) and old-old adults (aged 80 and older), and (c) young-old adults residing in Taiwan compared to young-old adults in Germany.

## Study 1: Psychometric Properties of the AARC Short Form in Taiwan

### Method

#### Participants and procedures

The study is based on analyses of cross-sectional data collected through the ongoing Healthy Aging Longitudinal Study in Taiwan (HALST; Hsu et al., 2017). The study was approved by the IRB Committee of the National Health Research Institutes in Taiwan (EC1020805-R1). Written informed consent was obtained from all individual participants included in the study. The baseline sample was collected between 2009 and 2013 with community-dwelling older adults aged 55 and above. The AARC-SF scale was added to the study protocol for the third wave of the study beginning in August 2020 in Miaoli, Taiwan. Three hundred seventy-eight participants responded to the AARC-SF from 2020 to 2021. Data of 86 participants were excluded because the responses from the home interview were from their proxies rather than the adults themselves. This resulted in a final sample size of 292 adults (Supplementary Figure 1). Table 1 summarizes the sample characteristics of sociodemographic status, health status, and the main variables of interest, including the mean scores of gain- and loss-related changes derived from the AARC-SF. Seventy-one percent of the sample were young-old adults (*M* = 77.38 years, *SD* = 6.24 years), 52% were women, and with various levels of education (*M* = 10.56 years, *SD* = 9.11 years).

**Table 1.** Samples Description

Variables	Study 1	Study 2 (age range: 65–80 years)	
	Taiwan	Germany	Taiwan
	*n* = 292	*n* = 118	*n* = 207
Sociodemographic characteristics			
Age in years, mean (SD)^**^	77.38 (6.24)	71.12 (4.14)	74.26 (4.04)
Old-old (>80 years old), %	29.11	—	—
Women, %^*^	52	69	56
Education years, mean (*SD*)	10.56 (9.11)	11.51 (4.14)	10.79 (6.75)
Annual household income in US Dollar, %			
No income	4.3	0	2.9
USD$1–12,999	67.7	7.1	62.8
USD$13,000–19,499	6.4	36.7	8.1
USD$>19,500	5.9	56.2	12.0
Unknown	15.6	0	14.4
Subjective aging			
AARC-gains, mean (*SD*)	3.07 (0.87)	3.63 (0.59)	3.24 (0.83)
AARC-losses, mean (*SD*)	2.28 (0.86)	2.11 (0.58)	2.18 (0.81)
Proportional felt age, mean (*SD*)	−0.03 (0.09)	—	—
ATOA, mean (*SD*)	3.74 (0.94)	—	—
EBA, mean (*SD*)	2.85 (0.95)	—	—
Health-related variables		—	—
Chronic disease burden, mean (*SD*)	5.34 (2.4)	—	—
PCS, mean (*SD*)	49.87 (1.14)	—	—
MCS, mean (*SD*)	50.03 (1.01)	—	—
Self-rated health^a^ mean (*SD*)^**^	—	2.73 (0.81)	3.24 (1.01)

*Notes*: AARC = awareness of age-related changes; ATOA = attitude toward own aging; EBA = essentialist beliefs of aging; MCS = mental component score of the SF-12; PCS = physical component score of the SF-12; *SD* = standard deviation.

^a^Self-rated health ranged from 1 (*excellent*) to 5 (*extremely bad*); difference test between Taiwan versus Germany.

^*^ *p* < .05. ^**^p < .001.

## Measures

### AARC Short Form (AARC-SF) Taiwanese/Traditional Chinese Version

The 10-item AARC-SF scale assessed adults’ AARC in five behavioral domains. Each domain was assessed with two items, with one item describing a gain-related experience, and one item describing a loss-related experience. The stem for the questions was “With my increasing age, I realize that …,” and the responses were on a five-point Likert scale from 1 *= not at all* to 5 = *very much.* Mean scores of the AARC-gains and AARC-losses were calculated, with higher scores indicating higher levels of self-perceived age-related gains or losses. A rigorous translation/back-translation process by four bilingual scholars in the United States and Taiwan was employed for the AARC-SF to ensure that the same meaning was conveyed in both English and Chinese. Prior studies reported sound reliability of the AARC-SF with the German and the U.S. older adult samples, McDonald’s omega (ω) = 0.72 for AARC-gains and α = 0.80 for AARC-losses (Kaspar et al., 2019).

### Global subjective views of aging measures

Measures of other SVOA constructs included felt age, ATOA, and the essentialist beliefs of aging scale (EBA; Weiss & Diehl, 2021). The measures of felt age and ATOA were selected to evaluate convergent validity, as both measures evaluate individuals’ SVOA with a reference to chronological age or their younger selves, similar to the question stem of AARC-SF, “With my increasing age, I realized that ….” EBA was chosen to investigate discriminant validity because this construct measures individuals’ generalized beliefs about the malleability of the human aging process (e.g., “To a large extent, a person’s age biologically determines his or her abilities.”). Reliability and validity of the felt age, ATOA, and EBA measures have been established in prior studies (e.g., Brother et al., 2017; Diehl et al., 2023), with Cronbach’s αs ranging from 0.62 to 0.69 with the U.S. older adult samples.

### Health-related variables

Self-reported chronic disease burden and health-related quality of life were used to examine the criterion validity of the AARC-SF. *Chronic disease burden* was operationalized as the number of chronic diseases checked on a medical diagnosis checklist, including hypertension, diabetes, heart disease, stroke, and cancer. A total of 12 chronic diseases were listed, and a higher number indicated greater chronic disease burden. *Health-related quality of life* was assessed with the 12-item Short Form survey (SF-12; Ware et al., 2002). The SF-12 assessed the impacts of physical and mental health on individuals’ everyday functioning. Scoring followed the standard SF-12 algorithms, yielding the physical summary score (PCS) and the mental summary score (MCS). Higher scores indicated better functioning in everyday life and being less affeted by physical and mental health constraints. The psychometric properties of the SF-12 were established in prior studies (e.g., Ware et al., 2002).

### Sociodemographic information

Participants’ demographic and socio-economic status were derived from a structured questionnaire collected in the third wave data collection.

### Analytical Strategy

#### Item characteristics and reliability

At the level of individual items, classic item analysis indices, such as item difficulty, inter-item and item-total correlations were calculated. Cronbach’s alpha (α) was used to assess the internal consistency of items that generated the sum scores of AARC-gains and AARC-losses. Due to restrictive assumptions for alpha (e.g., uncorrelated errors) and the fact that the value of alpha is a function of the number of items included, McDonald’s omega (ω) was also calculated.

#### Factor structure

Because a two-factor structure of gain-related (i.e., AARC-gains) and loss-related self-perceptions of aging (i.e., AARC-losses) was supported by most previous studies, this study used confirmatory factor analysis to test this two-factor model in the Taiwanese sample. In addition to the Chi-square statistic, which is sensitive to sample size, alternative goodness-of-fit indices, including the comparative fit index (CFI), Tucker–Lewis index (TLI), root-mean-square error of approximation (RMSEA), and standardized root-mean-square residual (SRMR), were used for evaluating model fit (Hu & Bentler, 1999). Missing item responses, though trivial with the current sample (<0.8%), were handled by maximum-likelihood (ML) estimation for continuous variables with Mplus v.8.0 (Muthén & Muthén, 2017).

#### Evidence of validity

Convergent and discriminant validity were evaluated by calculating correlations between AARC-SF and felt age, ATOA, and EBA. The correlation coefficients were expected to be higher with conceptually more similar SVOA constructs (i.e., ATOA and felt age) and lower with substantively less similar SVOA constructs (i.e., EBA). Criterion validity of the AARC-SF was evaluated by examining the correlations of AARC with chronic disease burden and health-related quality of life, PCS, and MCS. The correlation coefficients were expected to be in the small to moderate range. Potential nonlinear associations between AARC and health correlates were also explored.

## Results

### Item Characteristics

Item analysis statistics for the 10-item AARC-SF are displayed in Table 2. On average, most respondents responded “moderately” to age-related gain experiences (AARC-gains) and “not at all” to “a little bit” to age-related loss experiences (AARC-losses) across the five behavioral domains. Overall, the items indicative of age-related gains were endorsed more favorably by the participants compared to items indicative of age-related losses.

**Table 2.** Item Characteristics, Factor Loadings, and Reliability of AARC-SF With the Taiwanese Older Adult Sample (*N* = 292)

Domain/item	隨著年紀增長, 我感覺自己… With my increasing age, I realize that…	Item difficulty	Item-total correlation	Factor loading	Reliability	
		*M* (*SD*)	*r*		α	ω
AARC-gains					0.79	0.79
INT+	更珍惜人與人之間的關係, 也更能欣賞別人 I appreciate relationships and people much more	2.84 (1.16)	0.58	0.69		
PHYS+	更加注重我的健康 I pay more attention to my health	3.66 (1.08)	0.61	0.71		
COG+	有更多的經驗和知識來看待人與事 I have more experience and knowledge to evaluate things and people	2.94 (1.20)	0.55	0.64		
SC/SE+	更清楚知道什麼對我來說是重要的 I have a better sense of what is important to me	3.15 (1.25)	0.63	0.71		
LIFE+	有更多的自由按照自己想要的方式過日子 I have more freedom to live my days the way I want	2.79 (1.17)	0.48	0.53		
AARC-losses					0.82	0.82
INT−	自己越來越依賴他人的幫助 I feel more dependent on the help of others	1.91 (1.15)	0.58	0.64		
PHYS−	自己的精力更少了 I have less energy	2.56 (1.09)	0.69	0.79		
COG−	我的腦袋越來越不靈光 My mental capacity is declining	2.56 (1.05)	0.56	0.64		
SC/SE−	發現激勵自己去做一件事情變得更難了 I find it harder to motivate myself	2.09 (1.16)	0.60	0.66		
LIFE−	自己的活動受到限制 I have to limit my activities	2.29 (1.17)	0.65	0.73		

*Notes*: Factor loadings are standardized coefficients. + = positive aspect of AARC (gains); − = negative aspect of AARC (losses); AARC = awareness of age-related changes; INT = interpersonal relations; COG = cognitive functioning; LIFE = lifestyle and engagement; SC/SE = social–cognitive and social–emotional functioning; PHYS = health and physical functioning; *SD* = standard deviation.

The pairs plot displayed in Supplementary Figure 2 shows the distribution of the item responses with the density plots (diagonal), as well as the values of the item-to-item correlations (the upper triangle) and graphical representations (the bottom triangle). The corrected item-total correlation coefficients for both AARC-gains and AARC-losses items were strong, indicating that these items were able to give information regarding an individual’s standing on each subjective aging experience. Item-total correlations also indicated that the items had good internal consistency for the construct they were supposed to measure: all the gain-related items were moderately and positively associated with one another but not with most loss-related items.

### Reliability and Factor Structure

The AARC-SF demonstrated satisfactory internal consistency, with α = 0.79 for AARC-gains and α = 0.82 for AARC-losses (Table 2). McDonald’s omega (ω) also suggested the respective items for the factors of AARC-gains and AARC-losses were substantively homogenous, with ω = 0.79 and ω = 0.82 for AARC-gains and AARC-losses, respectively. The two-factor model fit the data well according to conventional fit criteria, χ^2^(34) = 78.80, *p* < .001, CFI = 0.96, TLI = 0.95, RMSEA = 0.06, SRMR = 0.03. The two-factor model also fit significantly better than the single-factor model, Δχ^2^ = 607.00, *p* < .001; ΔCFI = 0.51. Standardized factor loadings showed that all items loaded well on their corresponding factors (Table 2).

### Examination of Validity

The results partially supported the hypotheses of convergent and discriminant validity, such that higher AARC-gains and lower AARC-losses were associated with better ATOA and a younger felt age (Table 3). The magnitude of the correlations tended to be stronger for AARC-losses compared to AARC-gains. Specifically, correlation coefficients (|*r|*) ranged from 0.21, a small effect, to 0.52, a large effect, for AARC-losses, whereas the correlation coefficients for AARC-gains ranged from 0.12 to 0.27, all small effects. Regarding discriminant validity, the results were in the expected direction. That is, adults’ EBA scores were positively associated with AARC-gains, and negatively associated with AARC-losses.

**Table 3.** Convergent, Discriminant, and Criterion Validity of the AARC-SF Chinese Version (*N* = 292)

Validity indicator		AARC-gains			AARC-losses		
Convergent and discriminant validity		*r*	95% CI	*p*	*r*	95% CI	*p*
	Felt age	−0.20	[−0.29, −0.10]	<.001	0.21	[0.11, 0.30]	<.001
	ATOA	0.27	[0.17, 0.36]	<.001	−0.52	[−0.59, −0.44]	<.001
	EBA	0.12	[0.02, 0.22]	.018	−0.33	[−0.42, −0.24]	<.001
Criterion validity							
	Chronic disease burden	−0.05	[−0.15, 0.05]	0.333	0.30	[0.18, 0.37]	<.001
	PCS	0.17	[0.07, 0.26]	0.001	−0.43	[−0.51, −0.34]	<.001
	MCS	−0.05	[−0.15, 0.06]	0.375	−0.38	[−0.46, −0.28]	<.001

*Notes*: AARC = awareness of age-related changes; ATOA = attitude toward own aging; CI = confidence interval; EBA = essentialist beliefs of aging; MCS = mental component score of the SF-12; PCS = physical component score of the SF-12.

Examination of criterion validity showed that higher levels of AARC-gains were associated with better PCS, *r* = 0.17, a small effect, but not significantly associated with MCS or chronic disease burden. In contrast, AARC-losses demonstrated significant associations with all three health indicators, namely, higher chronic disease burden (*r* = 0.30), lower levels of PCS *r* = −0.43), and lower levels of MCS (*r* = −0.38, Table 3). These effects were moderate in size. The analyses of nonlinear associations, specifically quadratic relationships, were statistically nonsignificant.

## Study 2: Measurement Invariance Across Gender, Age Groups, and Countries

### Method

#### Participants

Two sets of the AARC-SF data from the Taiwanese adult sample were utilized to perform tests of MI across groups. The full data set of 292 participants’ data used for Study 1 was employed to test MI across gender and age groups. To mitigate potential age biases in AARC, a subsample of 207 (70.90%) older adults aged 65–80 were selected for cross-country MI analyses, with the same-aged counterparts from Germany.

The German AARC-SF reference data were derived from the fourth measurement occasion of a larger longitudinal study focusing on SVOA and well-being (Diehl et al., 2013). From an initial baseline assessment in 2012 involving 423 participants, 233 (55.08%) were followed up to complete the 10-item AARC-SF online in 2020. Data from a subset of 118 older adult Germans aged 65–80 were included in the MI analyses.

Overall, the Taiwanese sample was significantly older, *t*(432) = 5.75, *p* < .001, and reported significantly poorer health compared to the German sample, *t*(426) = 4.94, *p* < .001. Compared to the Taiwanese sample, there were more women in the German sample, χ^2^(1) = 8.59, *p = *.004. Descriptive statistics of the German sample and comparisons with the Taiwanese sample are shown in Table 1.

### Analytical Strategies

A series of multigroup confirmatory factor analyses (MGCFA) models was applied to test MI between women and men, young-old and old-old adults, and between Taiwanese and German adults aged 65 and older (Vandenberg & Lance, 2000). The age group categorization was based on Kaspar et al. (2019), where the researchers found that adults aged 80 and older may endorse certain items differently compared to younger adults, the reference group. Please see the Supplementary Material for the steps used to test different levels of MI. To evaluate the fit of a more restricted model compared to a less restrictive one, we used the Chi-square difference test and the change in CFI by.01 or greater to determine whether the more restrictive model had a worse fit than the less restrictive one (Cheung & Rensvold, 2001).

## Results

### Measurement Invariance Across Gender

Compared to the configural invariance model, the metric invariance model between men and women did not substantially decrease the model fit. The results indicated that the items corresponding to each the AARC-gains and AARC-losses functioned equally well for men and women. The scalar invariance model did not yield a significantly worse model fit, either (Table 4). This meant that men and women responded to the items of the AARC-gains and AARC-losses subscales in a similar way, and any differences observed in the group means were not due to measurement biases. Hence, comparisons of both observed composite scores and estimated factor means between men and women were permissible.

**Table 4.** Goodness-of-Fit Indices for Measurement Invariance Models Across Gender, Age Group, and Country

Measurement Invariance Model	χ^2^	*df*	*p*	CFI	TLI	RMSEA	SRMR	Δχ^2^	Δ*df*	*p*	ΔCFI
Gender											
Configural	147.04	68	<.001	0.94	0.92	0.08	0.05				
Metric	152.61	76	<.001	0.94	0.92	0.07	0.06	5.57	8	.695	0
Scalar	166.97	84	<.001	0.93	0.93	0.07	0.06	14.35	8	.073	0.008
Age group: young-old vs old-old											
Configural	111.78	68	<.001	0.96	0.95	0.06	0.05				
Metric	117.98	76	<.001	0.97	0.96	0.05	0.05	6.22	8	.622	0.007
Scalar	139.56	84	<.001	0.95	0.95	0.06	0.06	21.56	8	.005	0.051
Partial scalar^a^	130.83	80	<.001	0.96	0.95	0.06	0.06	12.85	4	.012	0.002
Country											
Taiwan vs Germany											
Configural	137.34	68	<.001	0.94	0.92	0.07	0.06				
Metric	162.56	76	<.001	0.93	0.91	0.07	0.08	25.22	8	.001	0.01
Partial metric^b^	143.97	72	<.001	0.94	0.92	0.07	0.07	6.63	4	.156	0.002
Partial Scalar^c^	152.63	76	<.001	0.93	0.92	0.07	0.07	8.66	4	.070	0.004

*Notes:*

^a^This partial scalar invariance model allows to freely estimate the intercepts of the four items belonging to AARC-gains (INT+, COG+, SC/SE+, LIFE+) and two items belonging to AARC-losses (INT−, SC/SE−) of the young-old and the old-old.

^b^All the factor loadings were constrained equal across Taiwan and Germany, except for the loadings of PHYS+ and COG−.

^c^This partial scalar model specified equality of the factor loadings of eight items included in partial metric invariance model and the intercepts of four items belonging to AARC-losses across Taiwan and Germany. All five items of AARC-gains and COG− were allowed to be freely estimated. AARC = awareness of age-related changes; CFI = comparative fit index; RMSEA = root-mean-square error of approximation; SRMR = root-mean-square residuals; TLI = Tucker–Lewis index.

### Measurement Invariance Across Age Groups

Compared to the configural invariance model, the metric invariance model did not show a significant loss in its model fit. Thus, the contributions of each item to the concepts of AARC-gains and AARC-losses appeared to be the same for young-old and old-old participants. However, the scalar invariance model yielded a significantly worse fit compared to the metric fit, suggesting that the assumption of equal item intercepts across age groups was not tenable.

Partial scalar invariance tests provided evidence of response shifts in four AARC-gains items related to interpersonal relations, cognitive functioning, social–cognitive and social–emotional functioning, and lifestyle and engagement when the latent AARC-gains value was set to 0 (Table 5). Substantial differences were also observed for two AARC-losses items related to interpersonal relations and lifestyle/engagement. Because of age biases in these items, old-old adults’ composite AARC-gains score may be underestimated, whereas their AARC-losses score may be overestimated.

**Table 5.** Measurement Parameters and Tests of Measurement Invariance Across Gender, Age Groups, and Countries

Domain/item	Factor loading			Intercept; mean (*SE*)		
	Age					
AARC-gains	Young-old	Old-old	Noninvariance	Young-old	Old-old	Noninvariance
INT+	0.73	0.62		3.03 (0.07)	2.57 (0.11)	x
COG+	0.60	0.63		3.14 (0.07)	2.67 (0.10)	x
PHYS+	0.74	0.71		3.77 (0.06)	3.56 (0.09)	
SCSE+	0.73	0.65		3.35 (0.07)	2.84 (0.11)	x
LIFE+	0.49	0.53		2.98 (0.07)	2.47 (0.09)	x
AARC-losses						
INT−	0.67	0.58		1.73 (0.06)	2.23 (0.11)	x
COG−	0.63	0.68		2.50 (0.06)	2.68 (0.09)	
PHYS−	0.76	0.84		2.49 (0.07)	2.67 (0.09)	
SCSE−	0.67	0.65		1.98 (0.07)	2.28 (0.10)	x
LIFE−	0.72	0.71		2.16 (0.07)	2.50 (0.10)	
	Country					
AARC-gains	Germany	Taiwan	Noninvariance	Germany	Taiwan	Noninvariance
INT+	0.46	0.68		3.43 (0.10)	2.98 (0.08)	x
COG+	0.59	0.59		3.72 (0.08)	3.10 (0.08)	x
PHYS+	0.23	0.74	x	3.28 (0.09)	3.80 (0.07)	x
SCSE+	0.75	.80		3.88 (0.08)	3.31 (0.08)	x
LIFE+	0.30	00.49		3.80 (0.10)	3.03 (0.08)	x
AARC-losses						
INT−	0.53	0.61		1.77 (0.08)	1.73 (0.06)	
COG−	0.36	0.65	x	2.20 (0.09)	2.50 (0.07)	x
PHYS−	0.79	0.79		2.46 (0.08)	2.47 (0.09)	
SCSE−	0.50	0.63		1.84 (0.07)	1.98 (0.08)	
LIFE−	0.83	0.70		2.31 (0.09)	2.21 (0.08)	

*Notes*: Factor loadings are standardized regression coefficients. + = positive aspect of AARC (gains); − = negative aspect of AARC (losses); AARC = awareness of age-related changes; COG = cognitive functioning; INT = interpersonal relations; PHYS = health and physical functioning; SC/SE = social–cognitive and social–emotional functioning; *SE* = standardized error.

### Measurement Invariances Across Countries

In comparing the AARC-SF between Taiwan and Germany, the configural invariance model yielded satisfactory fit (Table 4). However, the metric invariance model showed significantly poorer fit than the configural model, Δχ^2^(8) = 25.22, *p* = .001, ΔCFI = 0.01. Notably, the factor loadings of gains in physical health and losses in cognitive functioning were relatively larger for the Taiwanese group compared to the loadings of the other items, suggesting that Taiwanese older adults placed greater emphasis on the impacts of gains in physical health and losses in cognitive functioning when considering their AARC compared to German older adults. Partial invariance models confirmed this hypothesis; that is, after allowing the factor loading of gains in physical health and losses in cognitive functioning to vary between groups, the partial metric invariance model did not show a significantly worse fit than the configural model, Δχ^2^(4) = 6.63, *p* = .156, ΔCFI = 0.002. There was no evidence that any of the other factor loadings were problematic.

The full scalar invariance model demonstrated significantly poorer fit than the partial metric invariance model, indicating distinct response patterns to some items between the two groups who were assumed to have the same absolute level of AARC-gains and AARC-losses. Independent-samples *t*-tests revealed statistically significant differences in item scores related to AARC-gains between the two groups, with the Taiwanese group responding significantly lower in almost all items, except for gains in physical health. Conversely, the two groups did not show significant differences in item scores related to AARC-losses, except for losses in cognitive functioning (Table 5).

A follow-up step-by-step iterative process leading to the final partial scalar invariance model was employed to validate differences in item responses. When the constraints on all five-item intercepts related to AARC-Gains and AARC-Losses in cognitive functioning were lifted, the partial scalar invariance model did not exhibit significantly poorer fit than the partial metric model, Δχ^2^(4) = 8.66, *p* = .07, ΔCFI = 0.004. This suggested that, at the same latent level of AARC compared to German same-aged adults, the age-related gains of Taiwanese young-old may be underestimated in most of the behavioral domains, whereas their losses in cognitive functioning may be overestimated (Table 5).

## Discussion

Drawing on the theoretical concept of AARC and existing evidence regarding the psychometric properties of the AARC-SF scale, this study examined whether awareness of age-related gains and losses could be assessed in a reliable and valid way in Chinese-speaking older adults. Furthermore, this study examined MI across different social groups, encompassing gender, age, and countries. The findings further underscore the importance of accounting for age group- and country-specific influences in the interpretation and comparison of individuals’ subjective aging experiences.

### Psychometric Properties of the AARC-SF Taiwanese/Traditional Chinese Version

The results pertaining to the psychometric properties indicated that the Chinese version of the AARC-SF scale was adequate and reliable in assessing positive and negative age-related changes in five behavioral domains. This study also demonstrated convergent validity of the AARC-SF scale in the Taiwanese sample by showing that scores of both AARC-gains and AARC-losses were significantly associated with the unidimensional SVOA measures of felt age and ATOA. On the other hand, results regarding discriminant validity supported the study hypotheses; that is, higher awareness of age-related losses and lower awareness of age-related gains were associated with a stronger belief that changes with aging are biological and nonmalleable in nature.

Criterion validity was supported by cross-sectional associations of AARC-gains and AARC-losses with health-related variables, aligning with prior findings indicating the greater predictive relevance of AARC-losses for individuals’ health and well-being (Brothers et al., 2017). These results were not entirely surprising, though. When individuals are asked about aging in their day-to-day lives, what most likely comes to mind are losses and declines, especially losing functional ability or mobility, rather than gains in emotional and social well-being (Heckhausen et al., 1989).

### Measurement Invariance Across Gender, Age Groups, and Countries

Results indicated that the AARC-SF is suitable for comparing AARC-gains and AARC-losses between men and women. Establishing MI in this case provided a foundation for meaningful gender comparisons, uncovering nuanced distinctions between men and women in their perceptions of age-related gains and losses free from concerns of measurement bias.

Achieving scalar variance between young-old and old-old adults proved challenging as has been shown in several other studies (e.g., Kaspar et al., 2019, 2024). This challenge was not unexpected because individuals may experience aged-related changes that are intrinsically and qualitatively different at different stages of later adulthood. For example, a 65-year-old might ponder on potential losses in social status and interpersonal relations and more gains in freedom for life as they approach retirement, whereas this is not relevant anymore for a person who has been retired for years. Conversely, an 85-year-old may experience more pronounced declines in physical and cognitive functioning, while placing greater value on interpersonal relations, a phenomenon well documented as part of socioemotional selectivity theory (Carstensen et al., 2003). Consequently, the different life stages of older adults theoretically and empirically may result in different and meaningful response tendencies to specific items, resulting in higher or lower item means at the group level and noninvariance at the scalar level.

Cross-country comparisons revealed distinctions in the age-related experiences of older adults with different social and cultural backgrounds. Various factors, including item interpretations and mode of assessment, may contribute to these differences.

First, the physical health item “I pay more attention to my health” may potentially be interpreted differently in Taiwan, specifically the word “attention” in this context. In Taiwan, this item very likely carried some negative connotations and could well be considered as an item dedicated to AARC-losses instead of AARC-gains. That is, in Taiwan, health declines may promote more attention to individuals’ health and therefore this item may not capture the aspect of “paying attention”—taking more care of one’s own health. Concerns regarding the ambiguity of the physical health item have been noted in the Norwegian (Testad et al., 2022) and Korean (Nam & Kim, 2021) validations of the AARC-SF. To address this issue, a follow-up exploratory and confirmatory factor analysis may be conducted, interpreting this item either as part of age-related losses (AARC-losses) or replacing it with an item that is more clearly indicative of positive health-related changes. Such analyses would provide insights into the underlying assumptions and offer guidance on how to better handle the ambiguity of this item in these contexts.

Second, the consistent lower AARC-gains scores in the Taiwanese sample may also stem from a cultural context where older generations were socialized to be more reserved in their judgments and rather restrained in expressing subjective experiences and perceptions related to aging. This is supported by the more modest AARC-losses scores, the ATOA mean score, and a smaller discrepancy between actual age and felt age in the Taiwanese sample compared to the findings reported by Kaspar et al. (2019).

Additionally, a potential mode of administration effect may have contributed to these patterns. Prior research found that older adult participants tended to report less negativity when they had direct contact with interviewers compared to self-report situations (Luong et al., 2015). Given that in the HALST, the AARC-SF was administered by interviewers rather than by self-report (electronic self-administration in Germany due to the COVID pandemic), the mode of assessment in Taiwan may have influenced participants to respond in a more conservative direction. Further investigation is needed to determine whether these cross-country differences may be attributed to the administration method of the AARC-SF.

Although there is an ongoing debate regarding the necessity of full or partial scalar MI as a prerequisite for meaningful group comparisons (Robitzsch & Lüdtke, 2023), future comparative studies might explore potential adjustments to the items that exhibit distinct meanings and intercepts for the purpose of valid comparisons across age groups, time, and countries (Kaspar et al., 2024).

## Limitations

The study’s sample comprised healthier community-dwelling older adults from a single rural region in central Taiwan, limiting the generalizability of the findings to older adults who are not as healthy or who live in more urban areas of Taiwan. Similar concerns apply the comparisons between the Taiwanese and the German sample whose participants were recruited from a relatively affluent geographical area. It is important to note that any observed differences in AARC between the two countries could be influenced not only by cultural factors but also socio-economic factors.

Despite these limitations, the concise and multidimensional nature of AARC-SF allows for the substantive exploration of adults SVOA across various life domain while minimizes response burden on participants. From a practical standpoint, understanding the diverse subjective aging experiences across behavioral domains could inform public health policies regarding the areas where middle-aged and older adults may need extra support to optimize their SVOA (Sabatini et al., 2024). Similarly, applying the AARC-SF could be instrumental for large epidemiological studies that are interested in efficiently identifying middle-aged and older adults who may benefit from interventions aimed at promoting healthy aging. Thus, the AARC-SF provides a reliable and valid measure that can help in focusing resources and support on those who need them the most, thereby enhancing the overall effectiveness of public health initiatives to optimize healthy aging. Additionally, recognizing measurement noninvariance and differential item functioning across age groups and countries is imperative for ensuring valid longitudinal and group comparisons. This understanding, particularly from the cross-national MI results, is pivotal for future comprehensive investigations, examining country-specific and societal factors influencing SVOA.

## Supplementary Material

gnae086_suppl_Supplementary_Materials

## Data Availability

The data set utilized in this study has not been made publicly accessible to safeguard participants’ privacy and confidentiality. Requests for access to a deidentified version of the dataset are welcome and can be made to the corresponding author (HYT). All analyses were conducted using SPSS version 28.0.1.0. and Mplus v.8.0, and analytical code is available for replication purpose. The study design, hypotheses, and analytical plan were not preregistered.
