# Supplementary material for: Psychometric Properties and Measurement Invariance of the Awareness of Age-Related Change Short Form in Older Adult Samples From Taiwan and Germany
Source: Gerontologist. 2024 Jun 29;64(9):gnae086. doi: 10.1093/geront/gnae086 (PMC11308167; doi:10.1093/geront/gnae086)
Supplement: gnae086_suppl_Supplementary_Materials [file gnae086_suppl_supplementary_materials.docx]

**Supplementary Material**

**Study #2: Analytical Strategies**

Steps to test different levels of MI:

Step 1 tested the assumption of configural invariance where the same items were assumed to load on the same factors across groups. If the fit was not acceptable, this would imply that the AARC-SF items result in different factor structures in the different groups. Once the baseline configural model was confirmed, the parameter estimates of standardized factor loadings and item intercepts were presented for illustrative purpose.

Step 2 tested metric invariance, which evaluated whether the item loadings on the same factor (i.e., AARC-gains and AARC-losses, respectively) were identical in size across groups. If the fit was not worse than that of the configural model, it suggested that the items were functioning in identical ways across groups. If full metric invariance was not achieved, partial invariance, where certain item loadings were allowed to vary, was tested to understand which items bear different levels of significance for different groups.

Step 3 tested the assumption of scalar invariance, where the intercepts of the items were assumed equal across groups. The fit of this model was compared with the fit of the metric invariance model. Partial scalar invariance tests were implemented to understand differential item functioning. That is, whether and to what extent observed item scores are over- or under-estimated in one group relative to the other, assuming their same stance on the latent factor.

**Figure S1**

*Flow Chart of the Sample Selection for Data Analyses*

765 Participants in Miaoli city, Taiwan, were recruited to participate in the HALST baseline assessment from 2009 to 2010

86 (13%) Participants’ proxies responded to the AARC-SF; data from these cases were excluded from the current analyses

**292 (77%) Participants had self-reported data on AARC-SF for the current analyses**

464 (81%) Participants returned and were interviewed in the HALST third-wave assessment from 2020 to 2021

572 (88%) Participants returned and were interviewed for the HALST second-wave assessment from 2014 to 2015

378 (82%) Participants had data on AARC-SF

**Figure S2**

*Diverging Plot(s) of Item Responses*


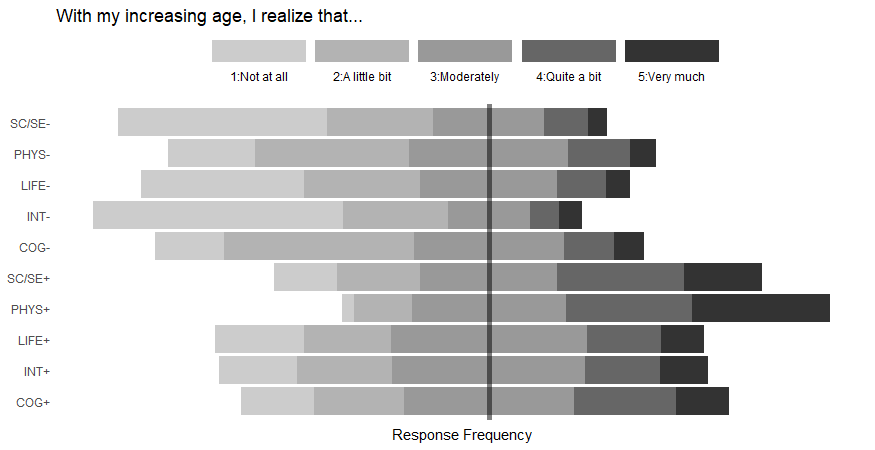


*Note.* INT = interpersonal relations; PHYS = health and physical functioning; COG = cognitive functioning; SC/SE = social-cognitive and social-emotional functioning; LIFE = lifestyle and engagement; + = positive aspect of AARC (gains); - = negative aspect of AARC (losses)

**Figure S3**

*Pairs Plot(s) of the AARC-SF Items*


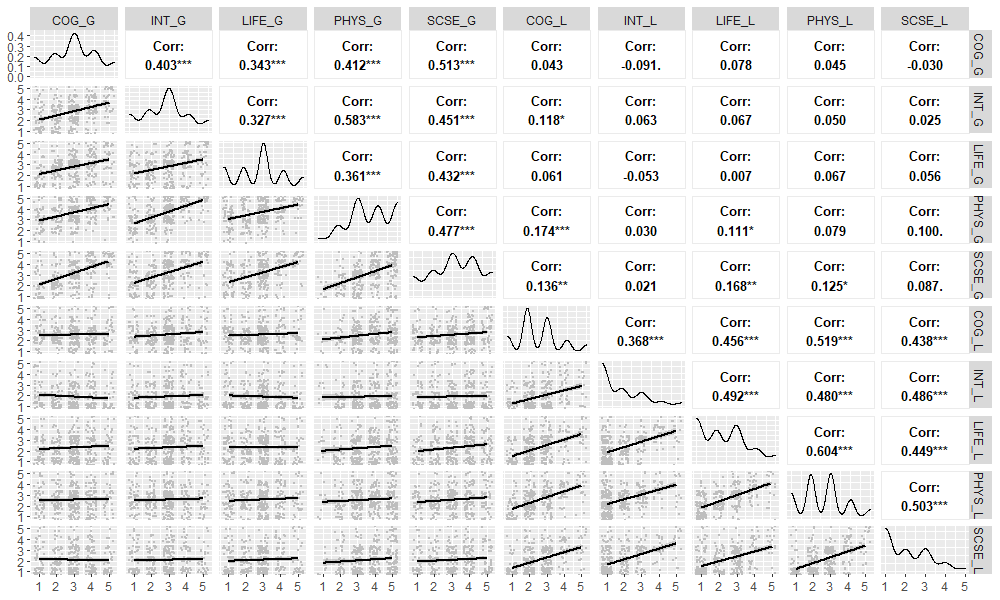


*Note.* COG = cognitive functioning; INT = interpersonal relations; LIFE = lifestyle and engagement; PHYS = health and physical functioning; SC/SE = social-cognitive and social-emotional functioning; _G = items representing AARC-gains; _L = items representing AARC-losses.

**Figure S4**

*Pairs Plot(s) of the AARC-SF with Unidimensional SVOA Indicators and Health-Related Variables (Top: AARC-losses; Bottom: AARC-gains)*


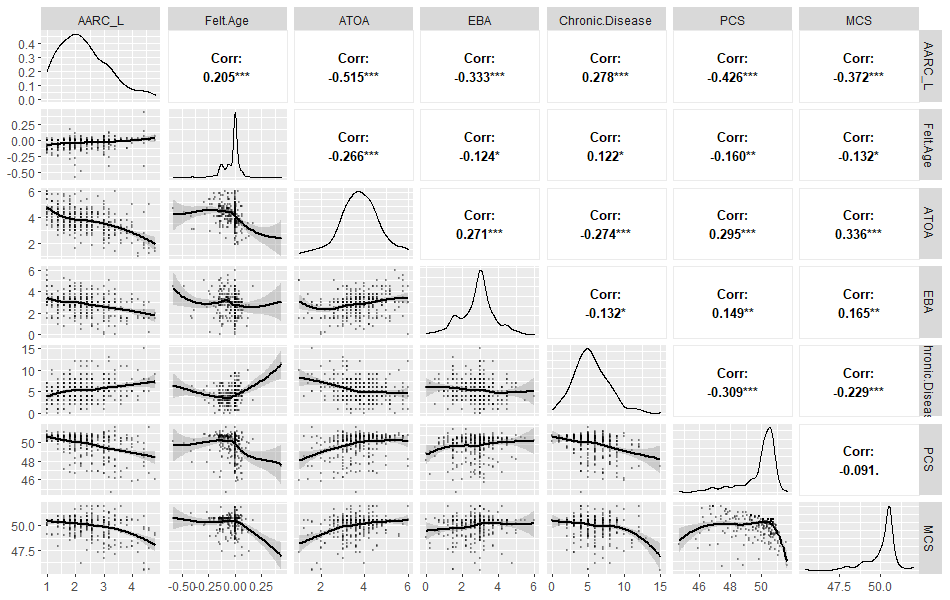

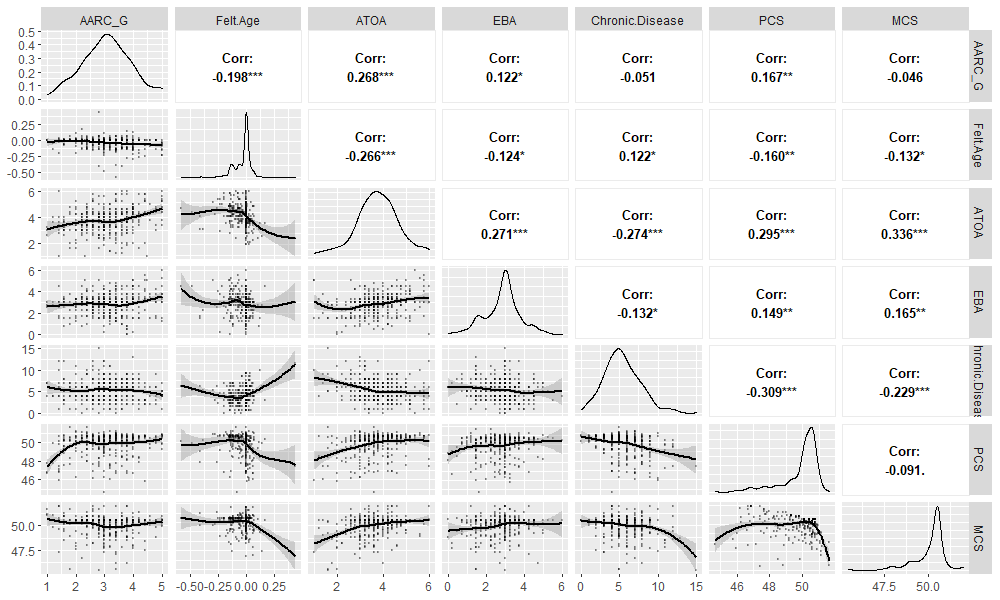


*Note.* AARC_G = mean score of five gain-related items representing the awareness of age-related gains; AARC_L = mean score of five losses-related items representing the awareness of age-related losses; ATOA = attitude toward own aging scale; EBA = essentialist beliefs of aging scale; PCS = physical component score of the SF-12; MCS = mental component score of the SF-12.
